# Comparison of remnant size in embolized intracranial aneurysms measured at follow-up with DSA and MRA

**DOI:** 10.1007/s00234-012-1063-3

**Published:** 2012-07-13

**Authors:** Zbigniew Serafin, Piotr Strześniewski, Władysław Lasek, Wojciech Beuth

**Affiliations:** 1 Department of Radiology and Diagnostic Imaging, Collegium Medicum, Nicolaus Copernicus University, ul. M. Skłodowskiej-Curie 9, 85-094 Bydgoszcz, Poland; 2 Faculty of Health Sciences, University of Humanities and Economics in Włocławek, Włocławek, Poland

**Keywords:** Intracranial aneurysm, Therapeutic embolization, Cerebral angiography, Magnetic resonance angiography, Follow-up studies

## Abstract

**Introduction:**

The possibility of recanalization and the need for retreatment are the most important limitations of intracranial aneurysm embolization. The purpose of the study was to compare the size of aneurysm remnants measured at follow-up with three-dimensional digital subtracted angiography (3D-DSA) and magnetic resonance angiography (MRA).

**Methods:**

Twenty-six aneurysms were found incompletely occluded in 72 consecutively examined patients at a follow-up after 3 months. The diameters and volume of aneurysm remnants were compared between 3D-DSA, time-of-flight MRA (TOF-MRA), contrast-enhanced TOF-MRA (CE-TOF-MRA), and contrast-enhanced MRA (CE-MRA) at 1.5 T.

**Results:**

There was a significant correlation between remnant volumes calculated based on 3D-DSA and all MRA modalities. The intraobserver variability of the measurements ranged from 3.4 to 4.1 % and the interobserver variability from 5.8 to 7.3 %. There were no significant differences in the variability between the techniques. The mean residual filling volume ranged from 16.3 ± 19.0 mm^3^ in TOF-MRA to 30.5 ± 44.6 mm^3^ in 3D-DSA (*P* < 0.04). Significant differences were found in the volumes measured with 3D-DSA and CE-MRA as compared to TOF-MRA and CE-TOF-MRA (*P* < 0.01). There was a moderate significant correlation between the residual filling and the relative error of measurement in the case of TOF-MRA and CE-TOF-MRA.

**Conclusions:**

TOF-MRA seems to underestimate the size of aneurysm remnants detected at follow-up and should not be used as a sole imaging method to decide on re-embolization.

## Introduction

Endovascular embolization has become a common method for the treatment of intracranial aneurysms in many centers of the USA and Europe [1, 2]. However, coiled aneurysms present a significant rate of recanalization, which occurs in approximately 20 % of patients [3, 4]. Due to the possibility of recanalization and the availability of a relatively safe endovascular retreatment [5], follow-up of coiled aneurysms is recommended [6, 7].

For many years, digital subtracted angiography (DSA) has been a method of choice for the follow-up of coiled aneurysms. However, several recent studies have proven a high diagnostic performance of magnetic resonance angiography (MRA) in detecting the incomplete aneurysm occlusion [8–15]. Therefore, due to a low invasiveness and a relatively low cost, MRA has been proposed as the first-choice modality for routine follow-up [16]. On the other hand, the question whether time-of-flight MRA (TOF-MRA) or contrast-enhanced MRA (CE-MRA) is a better method for the imaging of coiled aneurysms remains unresolved. In fact, both techniques present similar specificity and sensitivity in detecting the incomplete occlusion [8, 10], but CE-MRA offers better imaging of large aneurysm remnants and stented segments [9, 17].

Apart from detecting the incomplete aneurysm occlusion, follow-up imaging also aims at measuring the aneurysm remnant since the size of residual filling volume is the most important factor that determines the possibility of re-embolization [4]. However, technical differences between TOF-MRA and CE-MRA raise a question of the accuracy of residual volume measurements with these modalities. TOF-MRA is a flow-detecting modality which highlights blood flowing in limited directions above a threshold of velocity. Therefore, TOF-MRA may be unable to properly detect the actual volume of the residual aneurysm because of significant blood flow turbulence within the coil mesh.

The purpose of the study was to test the hypothesis that TOF-MRA is inadequate for measuring the residual filling volume in the embolized aneurysms and therefore should not be used for planning retreatment. Therefore, we compared the volume of the residual filling in the coiled aneurysms measured at follow-up with the use of three-dimensional DSA (3D-DSA), TOF-MRA, contrast-enhanced TOF-MRA (CE-TOF-MRA), and CE-MRA.

## Methods

### Population

The study was based on a population of patients that participated in a prospective single-center study on the diagnostic value of DSA and MRA in the follow-up of ruptured intracranial aneurysms [17]. The study was approved by the university institutional review board. All participants provided written informed consent.

Between November 2009 and March 2011, a total number of 72 patients (24 men; mean age, 51.5 ± 12.4 years) with 72 aneurysms were prospectively included in the study. Those patients were scheduled for the first follow-up imaging at 3 months after endovascular treatment for subarachnoid hemorrhage caused by aneurysm rupture. Patients were excluded for the following reasons: (a) age under 18 years; (b) contraindications to MR imaging, including severe claustrophobia, ferromagnetic foreign bodies, or electronic implants; (c) the presence of neurosurgical clips; and (d) estimated glomerular filtration rate <60 mL/min per 1.73 m^2^. At the end of all the embolizations, the baseline status of the aneurysm was documented by two-dimensional DSA (2D-DSA) and 3D-DSA.

### Follow-up DSA technique

DSA was performed using a monoplane angiographic unit (Axiom Artis dTA, Siemens Medical Systems, Erlangen, Germany) by means of transfemoral catheterization. Iodinated contrast material (iopromide, Ultravist 300 mg-I/mL, Bayer Schering Pharma AG, Berlin, Germany) was administrated with a power injector through a 5-F catheter. The examination consisted of a four-vessel 2D-DSA and 3D-DSA of the aneurysm parent artery. The 3D-DSA imaging was based on rotational acquisitions, covering 200°, resulting in 122 two-dimensional source images in cine mode. The contrast agent was administered at a volume of 15 mL (5 mL/s) to carotid arteries and 8 mL (3 mL/s) to vertebral arteries. Images were analyzed on a dedicated workstation (Syngo XVP VA72B, Siemens AG, Berlin, Germany) using InSpace 3D software. Measurements were calibrated to the diameter of the diagnostic catheter. The following reconstruction parameters were used: voxel size, 0.57 mm; number of slices, 220; slice matrix, 512 × 512; kernel type, EE; reconstruction mode, MIP; and dual volume.

### Follow-up MRA technique

MR angiography was performed with a 1.5-T Signa HDx unit using an eight-channel HD Brain Coil (GE Medical Systems, Waukesha, WI, USA) within 24 h after DSA. The examination consisted of three acquisitions: TOF-MRA, CE-TOF-MRA, and CE-MRA. TOF-MRA was performed with a 3D TOF ASSET Multislab technique (TE = 2.7 ms; TR = 30 ms; flip angle, 20°; bandwidth, 31.25 kHz; effective voxel size, 0.7 × 0.8 × 0.6 mm). The same scanner settings were used for CE-TOF-MRA, which was carried out after a manual intravenous injection of 3 mL of gadobenate dimeglumine (Gd-BOPTA; 0.5 mmol/mL, MultiHance, Bracco Imaging Deutschland GmbH, Konstanz, Germany). The examination was completed with CE-MRA (TE = 1.5 ms; TR = 3.5 ms; flip angle, 25°; bandwidth, 83.33 kHz; effective voxel size, 0.8 × 0.9 × 1.1 mm) after power injection of 0.1 mmol/kg b.w. of Gd-BOPTA at 2 mL/s followed by saline flush.

Angiograms were evaluated with Advantage Workstation 4.4 and Volume Share 8.4.3 software (GE Medical Systems). The analysis included non-reconstructed images as well as MPR, MIP, and VR reconstructions.

### Image analysis

Examinations of all patients were anonymized and divided into separate sets of 3D-DSA, TOF-MRA, CE-TOF-MRA, and CE-MRA images, which were randomly coded in a dedicated PACS folder.

The size of aneurysm remnants was compared between 3D-DSA, TOF-MRA, CE-TOF-MRA, and CE-MRA in patients who presented the filling in all the examined modalities. The presence of the residual filling was any leakage of contrast medium into the aneurysm neck or the aneurysm sac (class 2 or 3 according to [18]). Two interventional neuroradiologists (Z.S, P.S.) with a 10-year experience in the field independently assessed the blinded examinations, being unaware of other imaging results of the patients. The mean value of measurements performed by both investigators was used as a measurement result. By comparing the results presented by both observers, the inter-observer variability was calculated. One investigator (Z.S.) performed a second blinded analysis to calculate the intraobserver variability of the results.

Three perpendicular dimensions (*a*, *b*, *c*) were measured on MIP reconstructions to determine the flow volume according to the formula for ellipsoid (*v* = 4/3 × *π* × *a* × *b* × *c*). Additionally, the comparison included the largest dimensions which are common indicators of flow progression and the smallest dimensions which determine the possibility of retreatment.

### Statistical analysis

The diameters and volumes were expressed as the mean values ± SD. The normality of the data was tested with the Shapiro–Wilk test. The intraobserver and interobserver variabilities of the measurements were calculated as follows: var = 2×|*x* − *y*|/(*x* + *y*) × 100 %, where *x* and *y* are the results of repeated measurements. The significance of the differences between the tested methods was tested with ANOVA for repeated measures and then with Wilcoxon signed-rank test for a direct comparison between methods.

Further analysis of the MRA modalities was related to the residual filling volume and was carried out with an assumption that 3D-DSA was a reference method. The relation between the DSA and MRA results was determined using a Pearson correlation coefficient (*r*_*v*_). The differences between the residual filling volume detected on 3D-DSA images and on TOF-MRA, CE-TOF-MRA, and CE-MRA images were given as a relative error: *e*_*v*_ = 2 ×|*V*_DSA_ − *V*_*x*_|/(*V*_DSA_ + *V*_*x*_) × 100 %, where *V*_DSA_ is the volume measured on the 3D-DSA image and *V*_*x*_ is the volume detected with the tested MRA method. A possible influence of the size of residual filling on the relative error was tested using Pearson’s correlation coefficient (*r*_e_). For this purpose, 3D-DSA filling volume values were natural log-transformed to reduce unilateral skewness of the data.

A *P* value <0.05 was considered significant. Statistical analyses were performed using MedCalc 11.6.0 (MedCalc Software, Mariakerke, Belgium) and Statistica 9 (StatSoft Inc., Tulsa, OK, USA).

## Results

Follow-up examinations revealed residual flow in 26 aneurysms (36.1 %, Table 1). All the patients who presented incomplete occlusion had embolization performed with a combination of bare platinum coils (GDC Detachable Coils, Boston Scientific, Natick, MA; Axium and Nexus, ev3 Corporate, Plymouth, MN; MicroPlex, MicroVention, Inc., Tustin, CA) and hydrogel coils (HydroCoil and HydroSoft, MicroVention). Neither balloon remodeling nor stent assistance was used for these procedures.

**Table 1:** Baseline characteristics of the included patients with percentages in parentheses

Characteristics		Value (%)
Aneurysm location	ICA	18 (69.2)
	ACoA/ACA	4 (15.4)
	MCA	2 (7.7)
	BA/VA	2 (7.7)
Largest aneurysm diameter	Small (≤5 mm)	7 (26.9)
	Medium (5.1–15 mm)	16 (61.5)
	Large (15.1–25 mm)	3 (11.5)
	Giant (>25 mm)	0 (0.0)
Sack-to-neck ratio	≤1.5	13 (50.0)
	1.6–2.5	11 (42.3)
	>2.5	2 (7.7)
Number of coils placed	≤3	9 (34.6)
	4–6	8 (30.8)
	>6	9 (34.6)
Result of embolization^a^	Class 1	18 (69.2)
	Class 2	8 (30.8)
	Class 3	0 (0.0)

The assessment of the result of embolization was performed with 2D-DSA and 3D-DSA, while all the measurements were based on 3D-DSA

^a^According to Roy et al. [18]

**Fig. 1:**
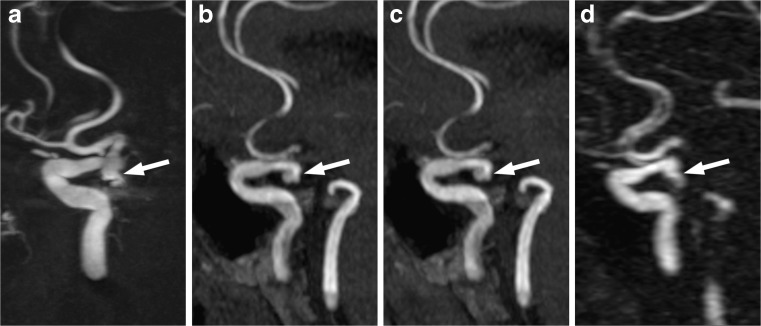
Residual aneurysm (*arrows*) of the left internal carotid artery presented on MIP reconstructions of 3D-DSA (**a**), TOF-MRA (**b**), CE-TOF-MRA (**c**), and CE-MRA (**d**)

**Fig. 2:**
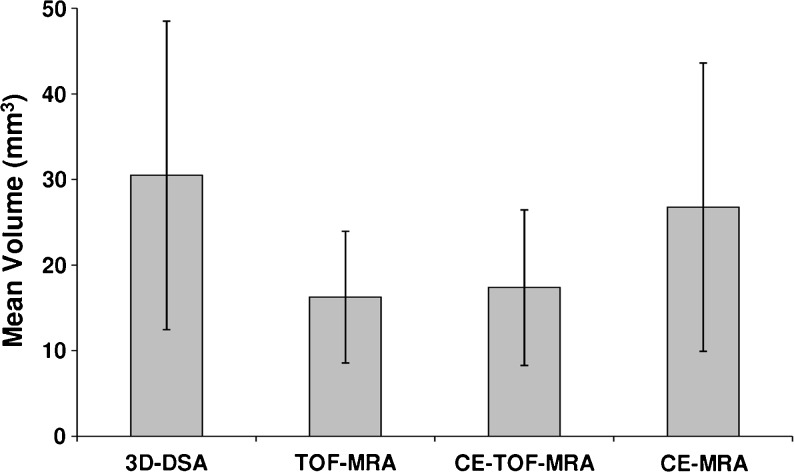
Mean volumes of aneurysm remnants with their 95% confidence intervals as measured on 3D-DSA, TOF-MRA, CE-TOF-MRA, and CE-MRA images

**Fig. 3:**
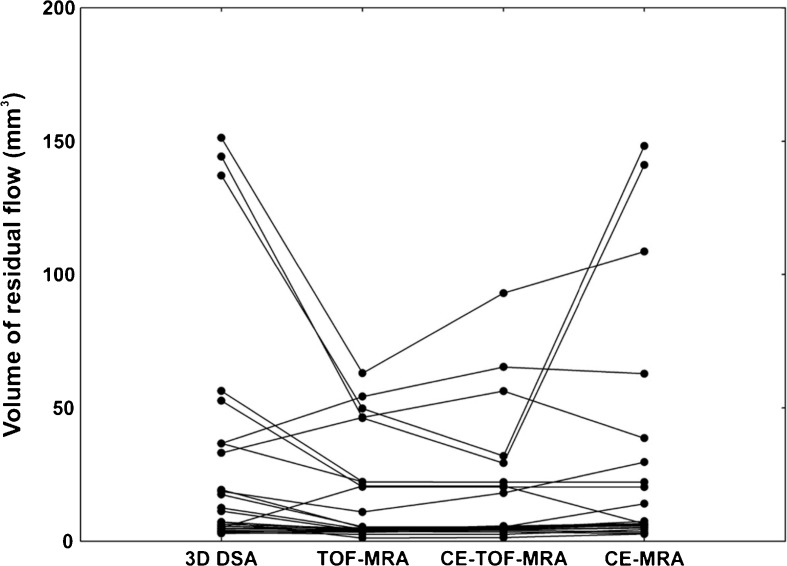
Direct comparison of the residual flow volume measured with 3D-DSA, TOF-MRA, CE-TOF-MRA, and CE-MRA in particular aneurysms

**Fig. 4:**
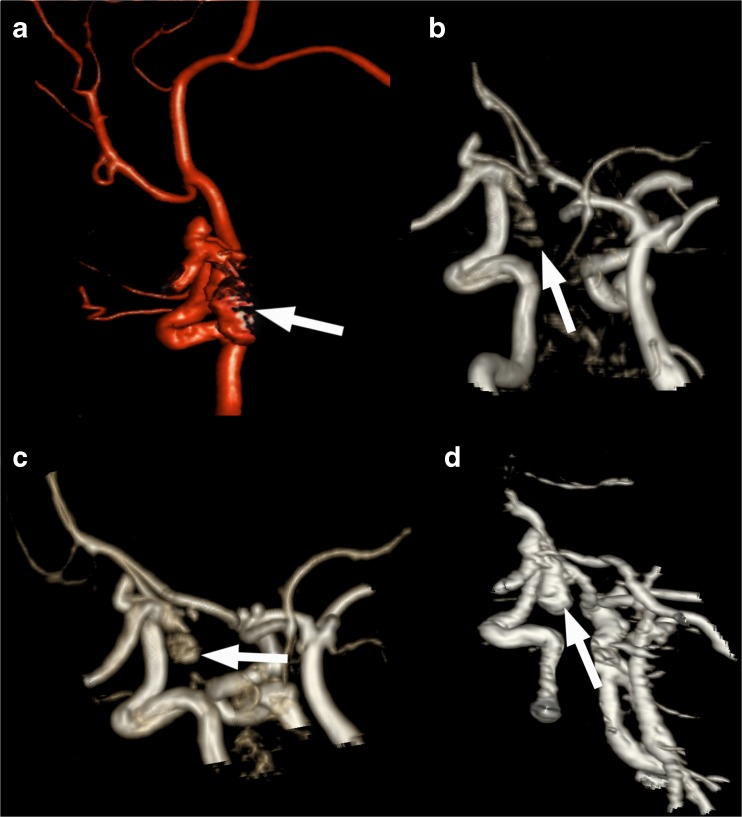
A case of recanalization of the right internal carotid artery aneurysm. The aneurysm remnant (arrows) has different sizes in 3D-DSA (**a**), TOF-MRA (**b**), CE-TOF-MRA (**c**), and CE-MRA (**d**)

The aneurysm remnants were classified as class 2 (residual neck) in eight cases and as class 3 (residual aneurysm) in 18 cases (Fig. 1). Compared to the initial results of embolization, recanalization of a totally occluded aneurysm was noted in 18 cases: class 2 in seven patients and class 3 in 11 patients. The progression of residual filling from class 2 to class 3 was observed in seven cases. There was also one stable aneurysm of class 2. Measurement of the flow area with the four tested angiographic methods gave significantly different results (ANOVA: *P* < 0.04; Table 2 and Fig. 2). The measures determined with the use of 3D-DSA and CE-MRA were significantly higher than those with TOF-MRA and CE-TOF-MRA (Wilcoxon signed-rank test: *P* < 0.01; Fig. 3). Follow-up DSA resulted in a decision for retreatment in 12 patients. A respective analysis of MRA images allowed for a positive decision on re-embolization in nine patients based on TOF-MRA and CE-TOF-MRA and in 11 patients based on CE-MRA. The most definite differences were seen in the case of large aneurysm remnants (Fig. 4). Intra-observer variability of the measurement was low, ranging from 3.4 to 4.1 %. Inter-observer variability ranged from 5.8 to 7.3 %. There were no significant differences in the variability between the techniques.

**Table 2:** Comparison of the largest diameter, the lowest diameter, and volume of the aneurysm remnants between the tested angiographic methods (mean values ± SD)

	Largest diameter (mm)	Lowest diameter (mm)	Volume (mm^3^)
3D-DSA	5.08 (±2.80)	2.26 (±0.97)	30.5 (±44.6)
TOF-MRA	3.73 (±2.09)	2.08 (±0.75)	16.3 (±19.0)
CE-TOF-MRA	3.86 (±2.21)	2.18 (±0.86)	17.4 (±22.5)
CE-MRA	4.36 (±2.42)	2.30 (±0.94)	26.8 (±41.7)

**Fig. 5:**
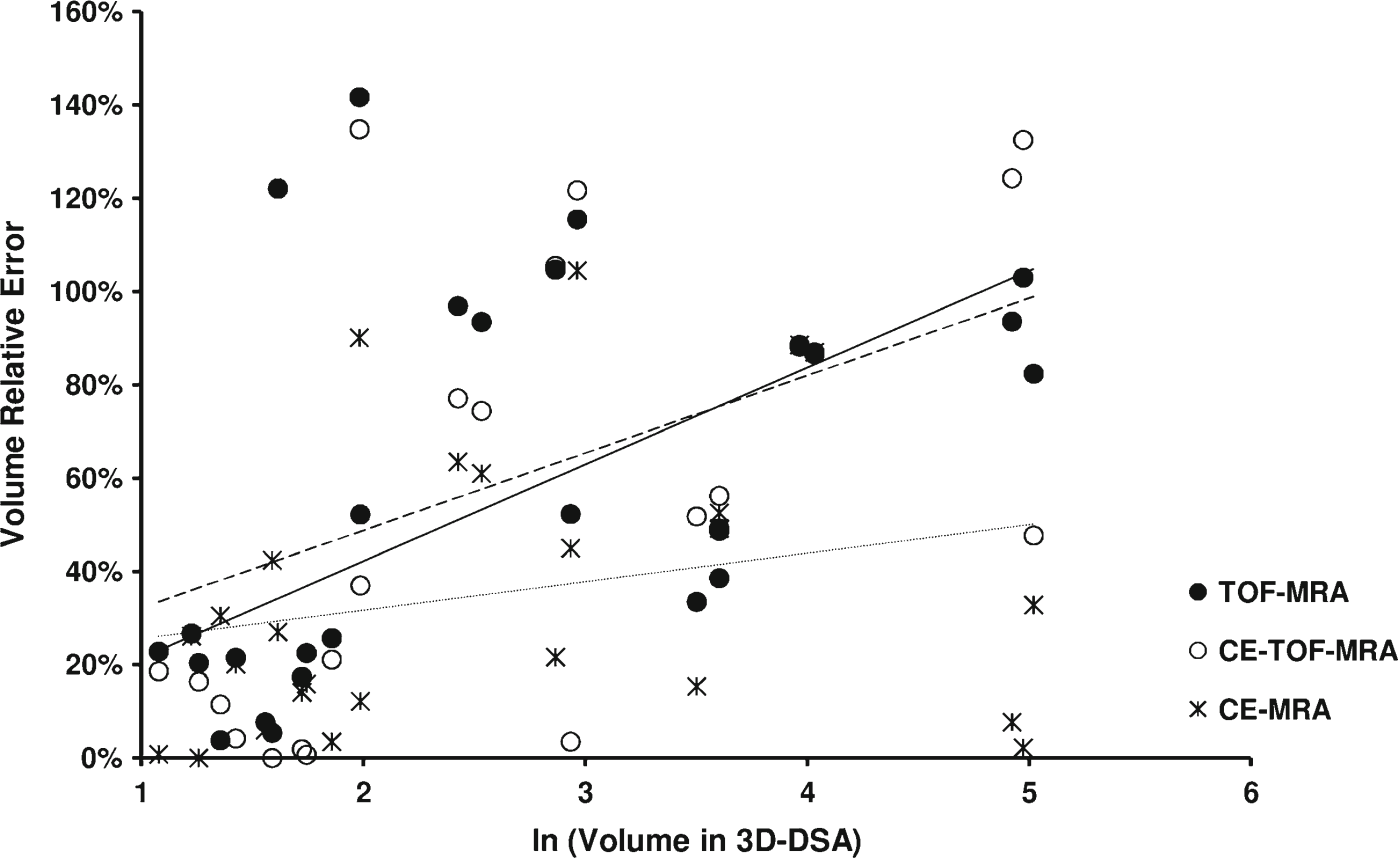
Relation between the volume of aneurysm remnants measured with 3D-DSA and the relative error of volume measurement with TOF-MRA, CE-TOF-MRA, and CE-MRA. Aneurysm remnant volumes were natural log-transformed in order to reduce unilateral skewness of the data

There was a significant correlation between the residual filling volume calculated based on 3D-DSA and all MRA modalities (*P* < 0.001). The highest correlation was noted between 3D-DSA and CE-MRA (0.95; Table 3). Correspondingly, CE-MRA had the lowest mean relative error of volume measurement, while TOF-MRA and CE-TOF-MRA presented similar values (Table 3). There was a moderately significant correlation between the residual filling volume and the relative error of measurement in the case of TOF-MRA and CE-TOF-MRA (Table 3 and Fig. 5).

**Table 3:** Analysis of the relation between the volume of aneurysm remnants measured with 3D-DSA and the three tested MRA methods

	*r*_*v*_	*e*_*v*_ (%)	*r*_e_
TOF-MRA	0.83	58.8 (±41.6)	0.50^b^
CE-TOF-MRA	0.69	54.7 (±46.9)	0.55^b^
CE-MRA	0.95	35.4 (±30.9)^a^	0.25

The relation is expressed as volume correlation (*r*_*v*_), relative error of volume measurement (*e*_*v*_), and correlation between the reference volume and relative error (*r*_e_)

^a^Significantly different from TOF-MRA and CE-TOF-MRA (*P* < 0.05)

^b^Statistically significant (*P* < 0.05)

## Discussion

According to our knowledge, this is the first clinical study to confirm that TOF-MRA at 1.5 T is inadequate for measuring aneurysm remnants. We found that the size of residual filling space measured with TOF-MRA and CE-TOF-MRA was significantly smaller than that measured with 3D-DSA and CE-MRA. These differences were most pronounced in the case of large aneurysm remnants.

In a recent study by Daugherty et al. [19], a significant variability among experienced operators was demonstrated when deciding whether a series of aneurysms should be retreated. The inter-reader discrepancies, which might have been substantial for patient management, were found in as many as 63 % of cases. Therefore, there is a need for strict criteria for the decision making on retreatment. One of such parameters may be the size of the residual flow area of at least 2 mm, which provides an appropriate space for an additional placement of the smallest coils [4]. Other proposed indications for retreatment include growth of an aneurysm remnant caused by coil loosening or coil compaction with unchanged aneurysm boundaries and aneurysm regrowth in increasing boundaries compared with the initial aneurysm size [20, 21]. However, with the use of a contemporary reference method of cerebral vessel imaging, i.e., 2D-DSA, a precise measurement of the aneurysm remnant is not an obvious issue. Despite the significant advantages of the DSA technology, it still offers linear measurements in pixels, not in millimeters, and requires calibration [22]. Therefore, precise measurements on 2D-DSA images require calibration on fiducial markers or diagnostic catheters. Another solution is the use of 3D-DSA, which has a finite isocenter and acquisition geometry, similar to those in CT and MR imaging that provide measurements in “real” millimeters [22].

Several studies proved the high diagnostic value of MRA in detecting aneurysm remnants; therefore, in many centers, MRA has become a first-line follow-up method after aneurysm embolization [8–15]. Once an aneurysm remnant is detected, it must be measured to determine possible progression and to decide on the need for retreatment. However, according to our results, the choice of follow-up method is essential. We found that the residual flow volume determined with the use of 3D-DSA and CE-MRA was significantly higher than those with TOF-MRA and CE-TOF-MRA. The mean volume of the flow area measured with the use of TOF-MRA was 87 % lower than in 3D-DSA and 64 % lower than in CE-MRA. Three-dimensional DSA is considered a method of choice for the detection, analysis, and planning of endovascular aneurysm therapy [17, 23]. It may also be considered the most precise modality of flow quantification since coils are selected for embolization based on 3D-DSA images without a need for any additional calibration. Thus, although TOF-MRA seems to be an accurate method for the detection of residual flow, it may not be considered a reliable method for flow quantification and for decision making on retreatment, which is a more important outcome of follow-up imaging.

The nature of TOF-MRA which detects blood flowing in limited directions above a threshold of velocity provides an explanation of the possible differences in filling volume quantification between the tested modalities. Therefore, TOF-MRA suffers signal loss in areas of slow and turbulent flow due to intravoxel dephasing and spin saturation [9]. The signal-loss effect may explain more evident differences in the volume of the aneurysm remnant in large-flow areas, as observed in our study. Moreover, the addition of a small amount of contrast medium during TOF-MRA acquisition did not result in a significant improvement of flow measurements. This indicates that the underestimation of the aneurysm remnant size in TOF-MRA is related to the time-of-flight technique itself rather than to the signal intensity. Although our results are quite obvious from the technical point of view, they seem to influence clinical practice significantly. If TOF-MRA replaces DSA as a standard method of follow-up to detect aneurysm incomplete occlusion, as proposed by several authors [16, 24, 25], in the case of positive results, it should be followed with CE-MRA or 3D-DSA to decide on possible retreatment.

There are limitations to this study that should be addressed. Firstly, the sample size was calculated for a different study that was aimed at determining the diagnostic value of DSA and MRA in the follow-up of intracranial aneurysms, and the 26 remnants found may be considered inadequate. Nevertheless, the presented differences in the residual flow size between the studied angiographic methods are statistically significant. Secondly, some measurement errors related to the 3D-DSA image reconstruction might have influenced our results because the flow size was dependent on individual window adjustment to some extent. However, the values of intra-observer and inter-observer variability were relatively low and did not differ significantly between the techniques. Moreover, none of our imaging methods was calibrated using dedicated phantoms. The study was designed to approach clinical practice, where the precision of measurements depends on the internal calibration of imaging devices and is controlled by routine quality assurance procedures. Finally, the study was performed with the use of a 1.5-T scanner. Application of a state-of-the-art 3-T system may provide different results because of the increased signal-to-noise ratio, spatial resolution, and contrast resolution [9].

## Conclusion

We found that in our limited patient population, the size of residual filling space in the embolized aneurysms measured at follow-up with 3D-DSA and CE-MRA was significantly higher than in TOF-MRA or CE-TOF-MRA. Therefore, in our opinion, TOF-MRA and CE-TOF-MRA may underestimate the size of the residual flow. In concordance with several previous reports, we still consider TOF-MRA as a first-line modality for follow-up to detect the aneurysm recurrence mainly because of its low invasiveness and the limited rate of aneurysm retreatment. However, once the recurrence is found, we suggest referring to CE-MRA of 3D-DSA to quantify the filling space, while a definite decision on re-embolization may be made based on 3D-DSA. The design of the study did not allow for the investigation of possible sources of the observed differences. Future research based on a larger patient population should be performed using automatic aneurysm segmentation and analysis systems, which have been proposed for both the 3D-DSA [26] and MRA [27].
